# Transforming mental health systems: The role of embedded researchers in advancing learning health systems

**DOI:** 10.1002/lrh2.70021

**Published:** 2025-07-18

**Authors:** Miranda Field, Christine Mulligan, Nicole D'souza, Raegan Mazurka

**Affiliations:** ^1^ University of Regina & Saskatchewan Health Authority Regina Saskatchewan Canada; ^2^ University of British Columbia & Foundry Vancouver British Columbia Canada; ^3^ University of Toronto & Jack.org Toronto Ontario Canada; ^4^ Dalhousie University & Nova Scotia Health Halifax Nova Scotia Canada

**Keywords:** embedded researchers, health system transformation, learning health systems, mental health systems

## Abstract

**Background:**

This commentary explores the critical role of embedded researchers in advancing Learning Health Systems (LHS) within the context of Canada's mental health systems.

**Context:**

The Canadian Mental Health Association has highlighted worsening mental health conditions, gaps in care, and disparities in access and outcomes.

**Approach:**

LHS offers a promising approach to address system challenges by transforming data into practical knowledge to drive continuous and rapid improvement. However, translating this vision into practice remains a challenge.

**Commentary:**

As four researchers currently embedded within the mental health system, working within public, nonprofit, and community settings, we argue that embedded researchers are an essential but often overlooked component of the workforce needed to implement LHS and improve mental health care. Embedded researchers, situated directly within the mental health sector, leverage their proximity to decision‐makers, knowledge users, and communities to bridge the gap between research, practice, and policy.

**Conclusion:**

This paper discusses the unique contributions of embedded researchers in driving systemic change, particularly within the three phases of the LHS cycle: data‐to‐knowledge, knowledge‐to‐practice, and practice‐to‐data.

## 1 INTRODUCTION

### 1.1 Canada's mental health system crisis and the potential of learning health systems

The Canadian Mental Health Association recently reported that the state of mental health in Canada is worse than ever, emphasizing gaps in the mental health care system, limitations in measuring and capturing relevant data, and disparities in access and outcomes for equity‐deserving and hard‐to‐reach groups.^1^, ^2^ These challenges are compounded by the fact that mental health is not explicitly covered under the Canada Health Act, leaving each province and territory to mandate and fund services differently. Unlike other areas of health care that are more uniformly integrated and delivered under universal coverage, mental health care in Canada has developed as a fragmented patchwork.^3^, ^4^ Community‐based organizations have often had to fill critical gaps, making it especially difficult to build coordinated, data‐driven mental health systems. Learning Health Systems (LHS)—cyclical systems that transform data into practical knowledge to drive continuous and rapid improvement—present a promising framework to address these challenges.^5^, ^6^, ^7^ In theory, LHS offer a pathway to eliminate silos, foster collaboration, and catalyze the evidence‐based innovation needed to create an integrated and adaptive mental health system.^8^, ^9^ However, translating this vision into practice remains elusive.

### 1.2 The role of embedded researchers

There is growing momentum for change within the mental health sector, and embedded researchers are uniquely positioned to accelerate the development and implementation of LHS. Unlike non‐embedded researchers, who are often based in academic settings and work at a distance from the systems they study, embedded researchers operate directly within the health system. They are deeply integrated into organizations, building sustained relationships with stakeholders such as mental health care professionals, policymakers, and diverse communities.^10^, ^11^, ^12^ This direct engagement allows embedded researchers to align their work with organizational priorities, ensuring that at least 70% of their time is dedicated to addressing system needs, bridging research and practice, and fostering meaningful, evidence‐informed change.^13^ Embedded research is not intended to replace traditional academic research (i.e., non‐embedded), but rather to complement. While there can be overlap, the key distinction lies in purpose: non‐embedded research typically aims to produce broad, generalizable findings that advance scientific knowledge, whereas embedded research prioritizes context‐specific, community‐driven, and rapidly actionable insights tailored to the needs of a particular organization or population. Despite their significant potential to drive transformation, embedded researchers remain an underrecognized yet essential component in realizing LHS.

### 1.3 Advancing the quintuple aim through embedded research

Embedded researchers play a critical role in advancing the Quintuple Aim,^14^ a suite of potential outcomes of LHS: enhancing patient experience, improving population health, reducing costs, supporting provider well‐being, and addressing health equity (i.e., equity in access, outcomes and reduction of barriers to mental well‐being).^15^ We use examples throughout this article to illustrate how embedded researchers can move the needle on the health equity component while simultaneously benefiting all target areas of the Quintuple Aim.

### 1.4 Our perspective

As four Canadian Institutes of Health Research (CIHR) Health System Impact fellows,^13^ embedded as researchers within the mental health sector—working within public, nonprofit, and community settings—we offer a real‐life perspective on how embedded researchers contribute to the three phases of the LHS cycle: data‐to‐knowledge, knowledge‐to‐practice, and practice‐to‐data. These interconnected phases represent a continuous learning process, where data from real‐world care is analyzed to generate insights, those insights are applied to improve practice, and the outcomes of those changes are then fed back into the system to inform further learning. Embedded researchers play a critical role in enabling and sustaining these cycles, helping mental health systems become more adaptive, evidence‐informed, and responsive to community needs. For example, by co‐designing evaluation tools with frontline youth workers to assess peer‐led mental health programs in real‐time, or by facilitating feedback loops between inpatient psychiatric units and outpatient providers to reduce readmission rates and improve continuity of care.

While the concepts of the LHS and embedded research have been explored from a broad health systems perspective,^16^, ^17^ at this inflection point in the mental health sector, there is a specific need to highlight how embedded researchers can—and should—be leveraged to drive mental health systems change. In the sections that follow, we explore the unique contributions embedded researchers make to each phase of the LHS cycle, demonstrating their value in advancing the Quintuple Aim and transforming mental health.

## 2 DATA‐TO‐KNOWLEDGE: HARNESSING MENTAL HEALTH DATA FOR IMPROVEMENT

Data is a cornerstone of LHS, but its value depends on its ability to inform decisions that lead to practical improvements.^18^, ^19^ To achieve this, raw data must be transformed into clear, practical insights—or “knowledge”—for decision‐makers. While many mental health organizations routinely capture large amounts of data—such as user demographics, service use statistics, and outcomes—much of this data remains underutilized. Embedded researchers, equipped with interdisciplinary methodological training, critical thinking, and subject matter expertise, can help organizations unlock the full potential of their data to address unique, organization‐specific challenges.

For example, a provincial or territorial health authority might be concerned about inefficiencies in mental health care delivery, such as duplication of services or gaps in care. While traditional metrics, like the number of people accessing care, provide a snapshot of service reach and growth, they can obscure underlying inequities, such as lower access among rural populations or equity‐deserving groups. These metrics alone are insufficient to identify the root causes of such disparities or to develop solutions tailored to the specific needs of the organization and the populations it serves.

Embedded researchers address these data‐to‐knowledge gaps by conducting deeper investigations and interpreting data beyond routine metrics, bringing dedicated time and adding capacity to the health system to make such in‐depth analyses possible. They may collect additional data, such as through key informant interviews, to uncover barriers like privacy concerns at home that hinder access to virtual care programs intended to reduce access inequities. Additionally, they may partner with interdisciplinary researchers to incorporate specialized data analysis techniques, such as geographic analyses, to better map service access and identify areas for improvement. Embedded researchers play a critical role in identifying and addressing biases in health system data and results by ensuring broad representation in the data collection process. By embedding principles of equity, diversity, and inclusion into research design and community engagement, they help reduce systemic biases and create more accurate, equitable, and responsive mental health systems.

This work is conducted in close collaboration with organizational decision‐makers, who drive the priority areas for investigation. By working to align data analysis with the organization's specific needs and strategic goals, embedded researchers ensure that the knowledge generated becomes a powerful tool for improvement of mental health services and equity of care, as in the above example, or in other target areas of the Quintuple Aim.

## 3 KNOWLEDGE‐TO‐PRACTICE: ADVANCING EQUITY AND FOSTERING TRUST

Embedded researchers, integrated directly within the mental health system and communities, can play a critical role in translating knowledge into practice. Unlike researchers from outside the system who engage intermittently on time‐limited, project‐based initiatives, embedded researchers dedicate their time to advancing organizational priorities across multiple projects over a significant time period. Their sustained presence allows them to build trust‐based relationships and form meaningful partnerships attuned to local contexts, creating a shared purpose of reducing health inequities through improved evidence‐based service provision. For instance, addressing the systemic inequities that persist in mental health service access and disproportionately affect Indigenous, racialized, 2SLGBTQI+ populations.

Beyond equity, embedded researchers contribute value to the knowledge‐to‐practice phase by improving the sustainability of interventions. Embedded researchers leverage their expertise in community engagement, knowledge mobilization, change management, and evaluation to seamlessly integrate evidence‐based practices into the operational fabric of health systems. By aligning initiatives with broader organizational goals, and adapting interventions to diverse contexts, they optimize resource allocation and ensure feasibility. Their day‐to‐day involvement provides a nuanced understanding of organizational and systemic constraints, enabling them to adapt evidence‐based interventions to fit the realities of service delivery. In contrast, non‐embedded researchers may produce generalizable findings but often leave the implementation of recommendations to other stakeholders, perpetuating the “knowledge to action gap”.^10^, ^20^ Embedded researchers also foster trust and engagement, addressing critiques of non‐embedded research as extractive or disconnected. By being woven into community organizations and collaborating to co‐design culturally relevant services, embedded researchers ensure interventions reflect lived experiences, respond to specific needs, and establish a foundation for equitable, sustainable practices. This makes them integral to mental health LHS.

## 4 PRACTICE‐TO‐DATA: CLOSING THE FEEDBACK LOOP FOR CONTINUOUS LEARNING AND ADAPTATION

Once knowledge is generated and practices are implemented, embedded researchers are critical to create feedback loops to enable continuous learning and systemic change, or practice‐to‐data. Embedded researchers do this by reviewing and synthesizing the latest evidence, translating complex findings into actionable insights, and tailoring them to the local context—ensuring that communities and practitioners can implement best practices without the burden of navigating the research themselves. For example, in a provincial youth mental health initiative, embedded researchers may identify that adolescents in hard‐to‐reach rural areas are frequently using emergency departments for mental health crises but have little access to consistent follow‐up care. Embedded researchers can then work with educators, social workers, and community leaders to co‐design a wraparound care model. This may include virtual counseling, school‐based mental health programs, and peer support networks tailored to the specific challenges faced by rural youth.

Real‐time data collection and analysis play a key role in an initiative like this, guiding continuous improvement. Embedded researchers can track service use, client satisfaction, and mental health outcomes, creating feedback loops that refine programs over time. If data reveal low engagement in virtual counseling among equity‐deserving youth, for example, researchers can then collaborate with community members to adapt the service, incorporating culturally responsive practices. These insights can then be shared with policymakers and pan‐Canadian networks to encourage broader improvements.

However, many mental health organizations, especially small nonprofits, lack the infrastructure to transform practice into actionable data to even start the LHS cycle. Without tools like standardized surveys to track outcomes and experiences, these organizations struggle to prioritize and improve services. Embedded researchers bridge this gap by supporting organizations to implement simple, scalable measures and translating data into actionable insights, ultimately benefiting the organization and the broader system. Embedded researchers can build capacity within the mental health system to measure what works and what does not, and then respond accordingly.

The practice‐to‐data process ensures that knowledge generated within organizations leads to both internal improvements and broader systems impact. Mental health intersects deeply with education, social services, and family supports, yet these sectors often work in isolation. Embedded researchers help break down these silos by fostering collaboration and transforming these fragmented systems into coordinated, adaptive networks. By sharing findings widely with intersectoral interest holders such as communities, researchers, and policymakers, embedded researchers promote equitable access to data and contribute to meaningful, inclusive, and lasting change.

## 5 EMPOWERING EMBEDDED RESEARCHERS TO ADVANCE LEARNING HEALTH SYSTEMS IN MENTAL HEALTH

Embedded researchers are vital to the realization of LHS in the mental health sector. Their ability to bridge research, practice, policy, and community partnerships ensures that each phase of the LHS cycle—data‐to‐knowledge, knowledge‐to‐practice, and practice‐to‐data—is effectively implemented, continuously refined, equitably, and sustainably executed. Furthermore, embedded researchers contribute directly to advancing the Quintuple Aim, enhancing patient experience, improving population health, reducing costs, supporting provider well‐being, and addressing health equity.

At the same time, integrating embedded researchers into health systems comes with challenges. These include the measurable costs of sustaining these roles, uncertainty around funding responsibilities, potential disruptions to existing organizational structures, and the reality that clinical teams already stretched to capacity may be hesitant to embrace additional change.^21^ Embracing embedded research and adopting a LHS approach also requires significant cultural change within an organization that requires sustained effort, motivation, leadership, and buy‐in.

To fully harness the potential of embedded researchers in the mental health system, actionable steps are essential:

### 5.1 Build sustainable infrastructure to support data‐to‐knowledge

Transforming raw data into actionable insights requires robust investment in data infrastructure. Mental health organizations must prioritize accessible, high‐quality data systems while providing training for staff to enhance data literacy. Embedded researchers can play a key role in supporting this capacity‐building. Policies should support the sustainable long‐term integration of embedded researchers into organizational decision‐making processes to ensure their analyses align with strategic priorities and address service inefficiencies and equity gaps.

### 5.2 Foster environments for transforming knowledge‐to‐practice

Applying evidence to practice demands environments that embrace collaboration, cultural humility, and inclusivity. Mental health organizations must invest in community engagement and knowledge mobilization, both within their own communities and across the broader mental health sector, to support system‐wide knowledge sharing. Ongoing professional development must be prioritized. While embedded researchers bring many skills, targeted training in areas like implementation science, change management, and evaluation can expand their capacity to drive sustainable, evidence‐based interventions. Investing in these opportunities enables embedded researchers to adapt to emerging challenges and maximize their impact within the system.

### 5.3 Enable practice‐to‐data feedback loops

For LHS to evolve, real‐time data from practice must inform future improvements. Organizations must invest in scalable tools such as standardized surveys or digital platforms to support smaller organizations that lack infrastructure. Additionally, embedded researchers should be positioned to lead cross‐sector collaborations, ensuring that insights from education, social services, and community networks are integrated into broader system improvements.

As LHS and the embedded research roles that support them continue to evolve in the mental health sector, it is essential to establish robust evaluation metrics. These metrics, which are currently emerging for both LHS and embedded research, are essential for assessing impact, identifying areas for adaptation, and ensuring continuous improvement in meeting the needs of the mental health system and its interest holders.^9^, ^22^, ^23^

## 6 OUR VISION

Looking ahead, we envision a future where embedded researchers are not the exception but a fundamental part of the mental health workforce, shaping how LHS adapt and deliver sustainable and equitable outcomes and experiences. We have seen firsthand the potential impact that embedded researchers can have on transforming how evidence is generated, applied, and sustained within the mental health sector.

No longer an overlooked or under‐supported role, embedded researchers will be positioned within organizations as key contributors to decision‐making, helping to transform data into actionable solutions that improve care delivery and reduce inequities. With sustainable funding mechanisms and collaboration from academia, government, and the nonprofit sector, embedded research can become a standard component of LHS, ensuring that mental health systems are not only efficient and accessible but also adaptive, evidence‐driven, and responsive to the evolving needs of the populations they serve. A truly transformative mental health system is one that learns and evolves; embedded researchers are the key to making this vision a sustainable, equitable, and lasting reality.

## FUNDING INFORMATION

All authors are current or former Canadian Institutes of Health Research (CIHR) Health System Impact Postdoctoral Fellows and received partial funding from CIHR as part of their fellowship agreements, which required co‐funding from health organizations. The first author was co‐funded by the Saskatchewan Health Research Foundation, the third author by Michael Smith Health Research BC and Foundry, and all other authors received co‐funding from their respective health organizations.

## CONFLICT OF INTEREST STATEMENT

The authors declare no conflicts of interest.

## USE OF AI STATEMENT

This writing is original in content and ideas, with an AI large‐language model used only for grammar corrections and clarification. The authors are aware it may flag AI indicators and openly declare how AI was used in this work.
